# Factors associated with PAs' choice of the traditional PANRE over the PANRE-LA

**DOI:** 10.1097/01.JAA.0000000000000411

**Published:** 2026-09-24

**Authors:** Andrzej Kozikowski, Mirela Bruza-Augatis, Tamara Ritsema, Cody Sasek, Yanlin Jiang, Andrew Dallas, Joshua Goodman

**Affiliations:** **Andrzej Kozikowski** is senior director of research; **Mirela Bruza-Augatis** is a senior research scientist; **Yanlin Jiang** is a senior measurement scientist; **Andrew Dallas** is director of psychometrics and data science; and **Joshua Goodman** is vice president of research and exam programs for the National Commission on Certification of Physician Assistants in Johns Creek, GA. **Tamara Ritsema** is an associate professor in the Department of Physician Assistant Studies at The George Washington University in Washington, DC. **Cody Sasek** is an associate professor and program director of the DMS Bridge Program at Butler University in Indianapolis, IN. C. Sasek is a clinical editor for *JAAPA*. The authors have disclosed no other potential conflicts of interest, financial or otherwise.

**Keywords:** recertification, longitudinal assessment, examination, PANRE, PANRE-LA, performance, physician associate

## Abstract

**Objective:**

To identify the factors associated with physician associates' (PAs') selection of the Physician Assistant National Recertifying Examination (PANRE) versus the Physician Assistant National Recertifying Examination–Longitudinal Assessment (PANRE-LA) for maintenance of board certification.

**Methods:**

We utilized data from 80,553 PAs due for recertification between 2024 and 2027 to examine associations between selecting the traditional PANRE versus the PANRE-LA and PA demographics, practice characteristics, prior traditional PANRE experience, most recent examination performance, and first-attempt Physician Assistant National Certifying Examination (PANCE) scores.

**Results:**

Overall, 77.6% of PAs chose the PANRE-LA. Selection of the traditional PANRE was most strongly associated with older age, previous experience with the traditional PANRE, male gender, and African American/Black race. Performance on the PANCE and the most recent traditional PANRE had a negligible association with PAs' choice of assessment type.

**Conclusion:**

Understanding the factors associated with examination choice is crucial for addressing PAs' current and future credentialing needs.

Since April 2023, all American Board of Medical Specialties (ABMS) member boards have offered their diplomates formative assessments for continuing certification.^1^ Many, including the American Board of Internal Medicine (ABIM), the American Board of Family Medicine (ABFM), and the American Board of Pediatrics (ABP), provide their diplomates with recertification options, including a traditional, proctored examination at a secure testing facility and a longitudinal assessment (LA).^2^-^4^ Approximately 80% of ABIM-certified physicians across disciplines have chosen the Longitudinal Knowledge Assessment over the traditional, long-form examination.^5^ Similarly, most ABFM diplomates (73%) and more than 80% of ABP diplomates opted for LAs over proctored examinations.^6^-^8^

Similar to many ABMS boards, the National Commission on Certification of Physician Assistants (NCCPA) offers two pathways for physician associates (PAs) to maintain their certification: the Physician Assistant National Recertifying Examination (PANRE) taken at a testing center and the Physician Assistant National Recertifying Examination-Longitudinal Assessment (PANRE-LA).^9^,^10^ Extensive research in cognitive psychology supports that LAs help maintain and update knowledge.^11^,^12^ More recent research conducted on newly developed LAs for continuing certification suggests that they are more flexible, reduce anxiety, and enhance learning and retention.^6^,^13^-^16^ A recent study examined PAs' initial perspectives on the PANRE-LA after they had completed the first two of the eight to 12 quarterly assessment recertification periods.^9^ More than 90% of PAs across diverse demographic and practice backgrounds indicated that the LA model enhances learning, identifies knowledge gaps, offers opportunities for improvement, supports lifelong learning, and keeps core medical knowledge up to date. Additionally, 84% felt it contributed to their growth as practitioners, whereas 78.7% had already applied or planned to apply what they learned from the PANRE-LA to their clinical practice.

Despite the advantages of LAs, many providers still select traditional assessments conducted in secure testing centers. There is limited research on the factors associated with providers' choices between LAs and traditional examinations. One study, however, found that physicians who chose to participate in the Family Medicine Certification Longitudinal Assessment (FMCLA) over the conventional, summative assessment were more likely to be younger and female and had lower scores on their most recent examination.^17^

Understanding the factors associated with examination choice can help better meet PAs' current and future credentialing needs. Given the limited research in this area, we explored whether PA demographics, specialties, experience, and performance on prior board examinations are associated with choosing the traditional PANRE over the PANRE-LA.

## METHODS

### Study design and population

This study employed a cross-sectional design with a population of 80,553 PAs who were due for recertification between 2024 and 2027, identified through NCCPA administrative records. The cohort included all PAs who could choose between the traditional PANRE and the PANRE-LA during this period to maintain their board certification. This study was deemed exempt from institutional review by the Sterling Institutional Review Board (IRB#13772).

### Data sources and variables

Data were obtained from NCCPA administrative records containing certification and examination history as well as the PA Professional Profile database, which includes demographic and practice characteristics.^18^,^19^ Data extraction was completed at the end of February 2024, capturing PA choices and attributes until that point.

The primary outcome of this study was PAs' choice of recertification examination format: the PANRE-LA or the traditional PANRE. The independent variables extracted from the NCCPA administrative records included whether PAs had previously taken the traditional PANRE, and, if so, what their most recent score was, as well as the score of their first attempt on the Physician Assistant National Certifying Examination (PANCE). Demographic variables obtained from the PA Professional Profile included age; gender; race; ethnicity; whether PAs speak another language in addition to English with patients; highest degree obtained; completion of postgraduate education; and rural-urban setting (classified using Rural-Urban Commuting Area codes). Practice characteristics included primary specialty (categorized as one of the top nine most common specialties or *other*; Table 1). Participants were asked to indicate what type of continuing medical education (CME) they most frequently completed (“What CME topics do you typically select the most?”). Response options included “topics related to my current specialty/practice area,” “topics related to core medical content,” and “other.”

**TABLE 1.** Characteristics of total PA cohort and by recertification examination choice

	Total cohort, N = 80,553	PAs who selected the PANRE-LA, n = 62,505 (77.6%)	PAs who selected the traditional PANRE, n = 18,048 (22.4%)	*P* value
**Gender**				<.001
Female	56,041 (69.6%)	45,782 (81.7%)	10,259 (18.3%)	
Male	24,499 (30.4%)	16,711 (68.2%)	7788 (31.8%)	
**Race**				<.001
White	63,931 (84.5%)	50,083 (78.3%)	13,848 (21.7%)	
Asian	4575 (6.0%)	3747 (81.9%)	828 (18.1%)	
African American/Black	2826 (3.7%)	1921 (68.0%)	905 (32.0%)	
Other^a^	4357 (5.8%)	3272 (75.1%)	1085 (24.9%)	
**Ethnicity**				<.001
Non-Hispanic/Latino(a)	71,008 (93.4%)	55,506 (78.2%)	15,502 (21.8%)	
Hispanic/Latino(a)	5015 (6.6%)	3797 (75.7%)	1218 (24.3%)	
**Bilingual**				<.001
No	59,245 (76.7%)	46,617 (78.7%)	12,628 (21.3%)	
Yes	17 995 (23.3%)	13,473 (74.9%)	4522 (25.1%)	
**Highest degree**				<.001
Master's	62,083 (78.1%)	50,652 (81.6%)	11,431 (18.4%)	
Doctorate	2338 (2.9%)	1769 (75.7%)	569 (24.3%)	
Other	15,087 (19.0%)	9331 (61.8%)	5756 (38.2%)	
**Postgraduate training**				<.001
No	67,256 (94.3%)	52,561 (78.2%)	14,695 (21.8%)	
Yes	4031 (5.7%)	2950 (73.2%)	1081 (26.8%)	
**Rural-urban setting**				<.001
Urban	74,128 (92.6%)	57,851 (78.0%)	16,277 (22.0%)	
Rural/isolated	5905 (7.4%)	4250 (72.0%)	1655 (28.0%)	
**Specialty**				<.001
Primary care^b^	16,499 (22.8%)	12,118 (73.4%)	4381 (26.6%)	
Surgery subspecialties	13,388 (18.5%)	11,046 (82.5%)	2342 (17.5%)	
Emergency medicine	8516 (11.8%)	6416 (75.3%)	2100 (24.7%)	
Internal medicine subspecialty	7151 (9.9%)	5651 (79.0%)	1500 (21.0%)	
Dermatology	3179 (4.4%)	2751 (86.5%)	428 (13.5%)	
Hospital medicine	2387 (3.3%)	1804 (75.6%)	583 (24.4%)	
Surgery-general	2135 (2.9%)	1738 (81.4%)	397 (18.6%)	
Psychiatry	1444 (2.0%)	1158 (80.2%)	286 (19.8%)	
Critical care medicine	1302 (1.8%)	1042 (80.0%)	260 (20.0%)	
Other	16,443 (22.7%)	13,091 (79.6%)	3352 (20.4%)	
**CME topics**				<.001
Current specialty	51,646 (68.1%)	41,058 (79.5%)	10,588 (20.5%)	
Core medical content	22,300 (29.4%)	16,490 (73.9%)	5810 (26.1%)	
Other	1879 (2.5%)	1338 (71.2%)	541 (28.8%)	

a Other races include American Indian/Alaska Native, Multiple races, Native Hawaiian/Pacific Islander, or other.

b Primary care includes family medicine, internal medicine-general, and pediatric-general.

### Statistical analysis

We first calculated descriptive statistics for all variables. Categorical variables were summarized using frequencies and percentages. Continuous variables, including PANCE and PANRE scores, were summarized using means and standard deviations. Bivariate analyses were conducted to examine unadjusted associations between each independent variable and the primary outcome of examination-type selection (traditional PANRE vs PANRE-LA). Associations involving categorical variables were evaluated using chi-square tests. Differences in continuous variables, including initial-attempt PANCE scores and most recent PANRE scores, between PAs who selected the traditional PANRE and those who selected the PANRE-LA were assessed using independent-samples t-tests. Analyses were performed for the overall cohort and stratified by age group (younger than 35 years, 35 to 44 years, 45 to 54 years, 55 to 64 years, and 65 years and older) and prior experience taking the traditional PANRE (yes, no). Finally, a multivariate logistic regression model was constructed to identify independent predictors of examination choice. Variance inflation factors were calculated to assess multicollinearity among the predictors in the model, all of which were between one and two, indicating that it was not an issue.^20^ All analyses were conducted using R version 4.2.3 (R Foundation for Statistical Computing, Vienna, Austria).

## RESULTS

**FIGURE 1.**
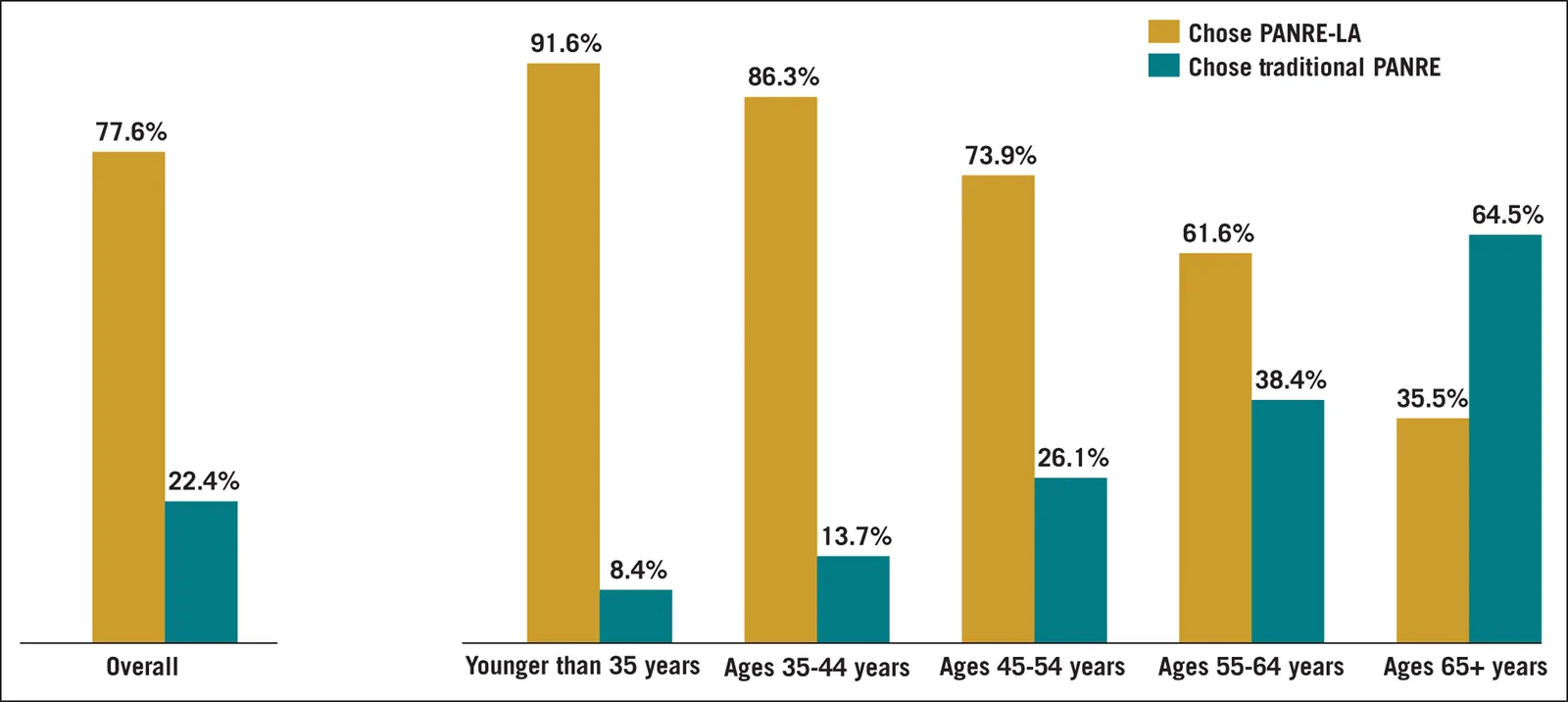
PA choice of PANRE-LA vs. traditional PANRE overall and by age groups∗

Most (77.6%) PAs chose the PANRE-LA, whereas 22.4% opted for the traditional PANRE. The likelihood of selecting the traditional PANRE, however, monotonically increased with age (*P* < .001; Figure 1). The majority of PAs age 65 years and older (64.5%) chose the traditional PANRE.

∗The association between age groups and recertification examination choice was statistically significant (*P* < .001).

Bivariate analyses revealed significant differences in examination choice across all assessed PA characteristics (all *P* < .001; Table 1). Notably, traditional PANRE selection was most common among PAs whose highest completed degree was below the master's level (PA certificate, associate, or bachelor's; 38.2% selected the traditional PANRE), those who identified as African American/Black (32.0%), and those who identified as male (31.8%). Conversely, traditional PANRE selection was least common among PAs practicing dermatology (13.5%) and surgery subspecialties (17.5%), those who identified as Asian (18.1%), and those who identified as female (18.3%).

Subgroup analyses examined PA characteristics based on prior experience with the traditional PANRE (Table 2). Among PAs without prior traditional PANRE experience (38.9% of the total sample), only 9.4% selected the traditional PANRE. Among those who selected the traditional PANRE, all demographic and practice characteristics, except ethnicity, postgraduate training, and CME topics, were significantly associated with examination-type selection. For those with prior traditional PANRE experience (61.1% of the total sample), 30.7% selected the traditional PANRE. Within this group, all demographic and practice characteristics were associated with examination-type selection (all *P* < .001). With the exception of ethnicity, postgraduate training, and CME topics, which differed in their associations with examination-type selection, the pattern of associations was similar across the two subgroups.

**TABLE 2.** Subgroup analyses of characteristics of PAs who had previously taken the traditional PANRE and those who had not

	Had not taken traditional PANRE previously, n = 31,319 (38.9%)			Had taken traditional PANRE previously, n = 49,234 (61.1%)		
	PANRE-LA, n = 28,388 (90.6%)	Traditional PANRE, n = 2931 (9.4%)	*P* value	PANRE-LA, n = 34,117 (69.3%)	Traditional PANRE, n = 15,117 (30.7%)	*P* value
**Gender**			<.001			<.001
Female	21,081 (92.7%)	1668 (7.3%)		24,701 (74.2%)	8591 (25.8%)	
Male	7307 (85.3%)	1263 (14.7%)		9404 (59.0%)	6525 (41.0%)	
**Race**			<.001			<.001
White	22,438 (91.1%)	2181 (8.9%)		27,645 (70.3%)	11,667 (29.7%)	
Asian	2047 (91.3%)	196 (8.7%)		1700 (72.9%)	632 (27.1%)	
African American/Black	746 (84.1%)	141 (15.9%)		1175 (60.6%)	764 (39.4%)	
Other^a^	1501 (90.2%)	164 (9.8%)		1771 (65.8%)	921 (34.2%)	
**Ethnicity**			.110			<.001
Non-Hispanic/Latino(a)	25,085 (90.9%)	2503 (9.1%)		30,421 (70.1%)	12,999 (29.9%)	
Hispanic/Latino(a)	1859 (89.9%)	210 (10.1%)		1938 (65.8%)	1008 (34.2%)	
**Bilingual**			.006			<.001
No	21,110 (91.1%)	2074 (8.9%)		25,507 (70.7%)	10,554 (29.3%)	
Yes	5965 (89.9%)	667 (10.1%)		7508 (66.1%)	3855 (33.9%)	
**Highest degree**			<.001			<.001
Master's	26,101 (91.0%)	2589 (9.0%)		24,551 (73.5%)	8842 (26.5%)	
Doctorate	569 (90.3%)	61 (9.7%)		1200 (70.3%)	508 (29.7%)	
Other	1263 (86.7%)	194 (13.3%)		8068 (59.2%)	5562 (40.8%)	
**Postgraduate training**			.181			<.001
No	23,318 (91.3%)	2235 (8.7%)		29,243 (70.1%)	12,460 (29.9%)	
Yes	1306 (90.2%)	142 (9.8%)		1644 (63.6%)	939 (36.4%)	
**Rural-urban setting**			.002			<.001
Urban	26,526 (90.8%)	2693 (9.2%)		31,325 (69.8%)	13,584 (30.2%)	
Rural/isolated	1696 (88.7%)	217 (11.3%)		2554 (64.0%)	1438 (36.0%)	
**Specialty**			<.001			<.001
Primary care^b^	5013 (89.3%)	603 (10.7%)		7105 (65.3%)	3778 (34.7%)	
Surgery subspecialties	5017 (92.3%)	418 (7.7%)		6029 (75.8%)	1924 (24.2%)	
Emergency medicine	3218 (88.4%)	422 (11.6%)		3198 (65.6%)	1678 (34.4%)	
Internal medicine subspecialty	2556 (92.5%)	206 (7.5%)		3095 (70.5%)	1294 (29.5%)	
Dermatology	1110 (94.7%)	62 (5.3%)		1641 (81.8%)	366 (18.2%)	
Hospital medicine	1021 (89.8%)	116 (10.2%)		783 (62.6%)	467 (37.4%)	
Surgery-general	836 (93.0%)	63 (7.0%)		902 (73.0%)	334 (27.0%)	
Psychiatry	652 (90.2%)	71 (9.8%)		506 (70.2%)	215 (29.8%)	
Critical care medicine	620 (91.3%)	59 (8.7%)		422 (67.7%)	201 (32.3%)	
Other	5684 (92.2%)	478 (7.8%)		7407 (72.0%)	2874 (28.0%)	
**CME topics**			.285			<.001
Current specialty	19,655 (91.1%)	1926 (8.9%)		21,403 (71.2%)	8662 (28.8%)	
Core medical content	6243 (90.5%)	656 (9.5%)		10,247 (66.5%)	5154 (33.5%)	
Other	411 (90.1%)	45 (9.9%)		927 (65.1%)	496 (34.9%)	

a Other races include American Indian/Alaska Native, Multiple races, Native Hawaiian/Pacific Islander, or other.

b Primary care includes family medicine, internal medicine-general, and pediatric-general.

Figure 2A illustrates the association between PA examination choice and first-attempt PANCE performance, both aggregated and within different age groups. Overall, PAs who chose the traditional PANRE scored slightly higher on their first attempt at the PANCE than those who chose the PANRE-LA (520.5 vs 512.6; *P* < .001). This pattern was consistent across age subgroups, except for in PAs younger than 35 years, where the difference was not statistically significant.

**FIGURE 2A.**
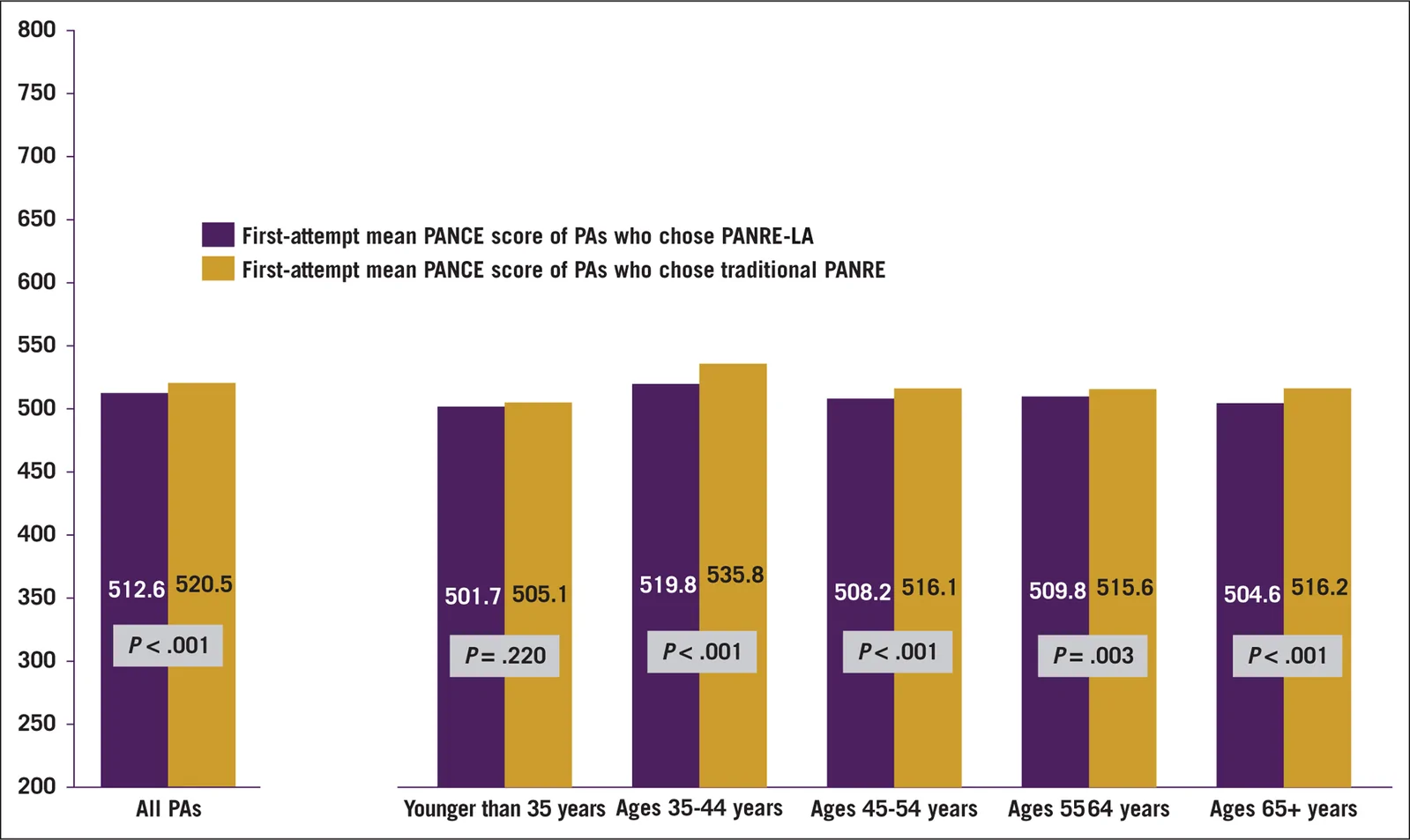
First-attempt mean PANCE scores by recertification examination choice of total PA cohort and within age groups

Figure 2B depicts the association between PA examination choice and performance on the most recent traditional PANRE, analyzed for the entire cohort as well as stratified by age group. A notable finding consistent with Simpson's paradox was observed: while the overall analysis demonstrated that PAs who chose the PANRE-LA had a significantly higher mean score on the most recent traditional PANRE compared with those who chose the traditional PANRE (509.1 vs 504.7; *P* < .001), the subgroup analyses by age group revealed an opposing trend.^21^ Within most age subgroups, PAs who selected the traditional PANRE demonstrated marginally higher mean scores. This reversal is attributable to age acting as a confounding factor. Traditional PANRE performance slightly declines with older age, and younger PAs, who tend to perform better, were more likely to select the PANRE-LA, thereby influencing the overall mean comparison (Figure 2B).

**FIGURE 2B.**
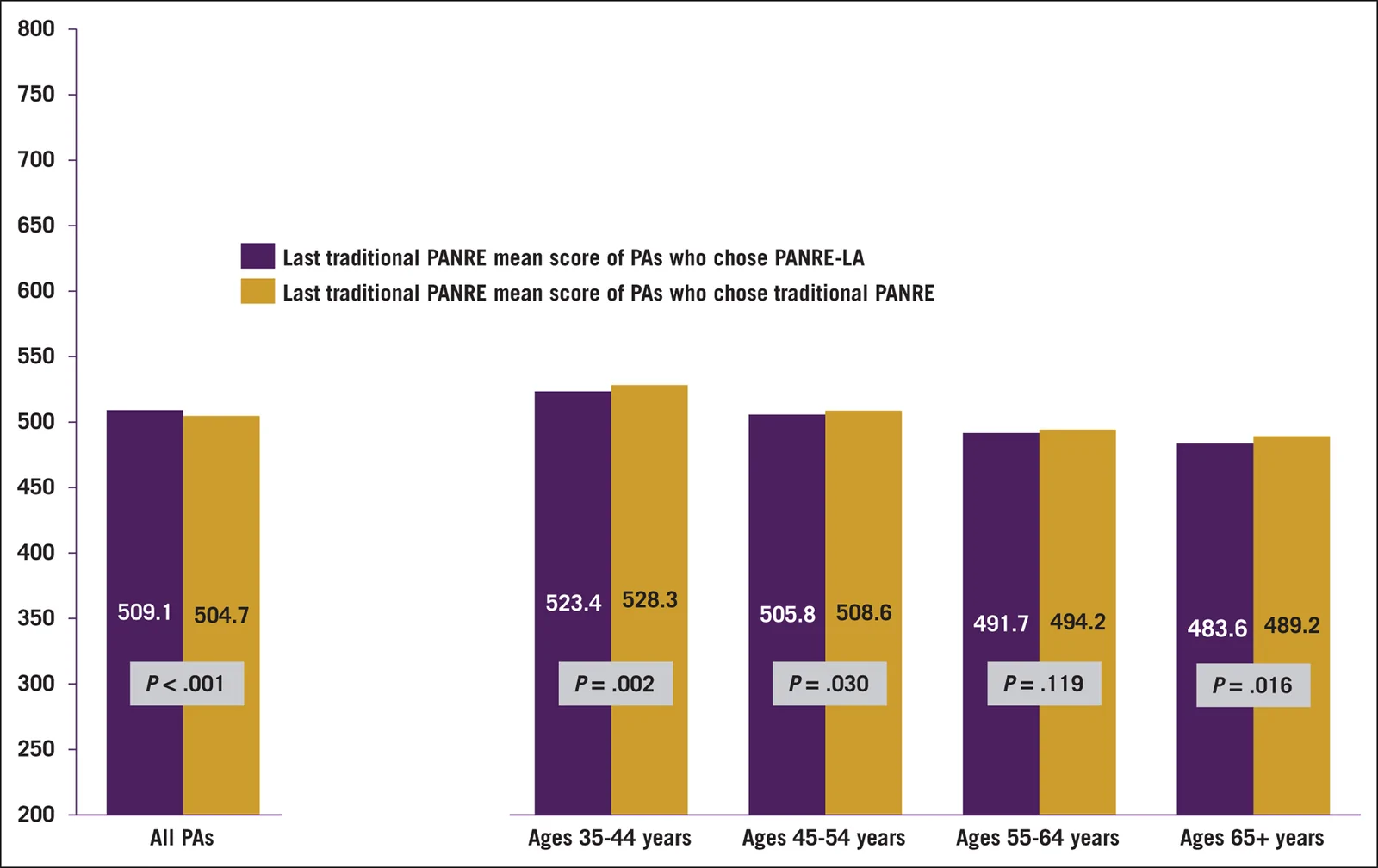
Last traditional PANRE mean scores of total PA cohort by recertification examination choice and within age groups∗

∗Age group younger than 35 years not included due to few PAs in this age group having previously taken traditional PANRE

**FIGURE 3.**
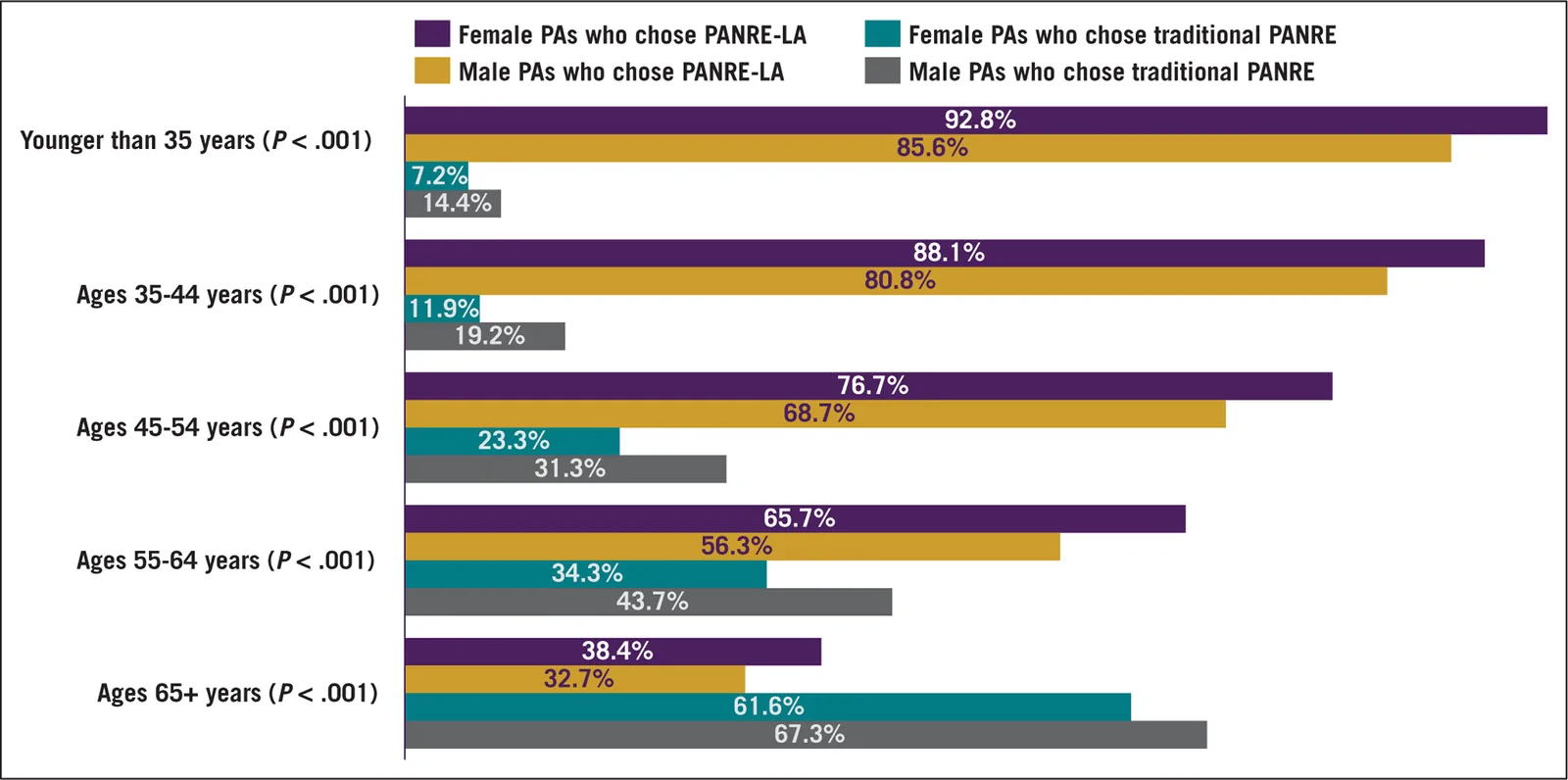
Recertification examination choices within age groups by gender

Figure 3 presents the results of PA examination choices within age groups, categorized by gender. In each age group, male PAs were more likely than female PAs to choose the traditional PANRE (all *P* < .001). The largest proportional difference between female and male PAs in choosing the traditional PANRE (34.3% vs 43.7%) was observed in the 55 to 64 age group.

**FIGURE 4.**
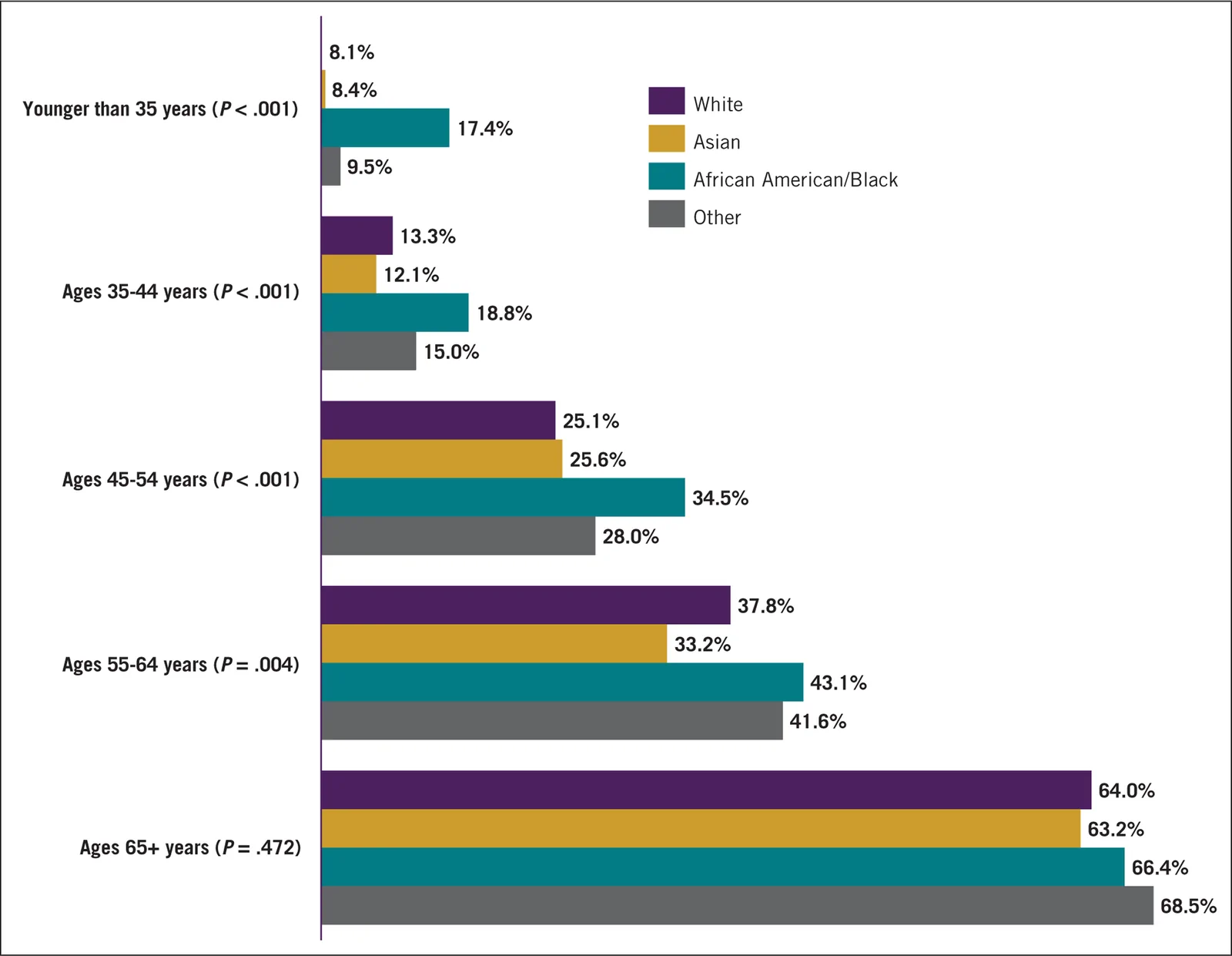
PAs who chose traditional PANRE within age groups by race

We also explored differences in examination choice within age groups by race (Figure 4). PAs who identified as African American/Black had a higher proportion selecting the traditional PANRE over the PANRE-LA than did other racial groups across all age subgroups (all *P* < .05) except among those ages 65 years and older, where the difference was not statistically significant (*P* = .472).

**FIGURE 5.**
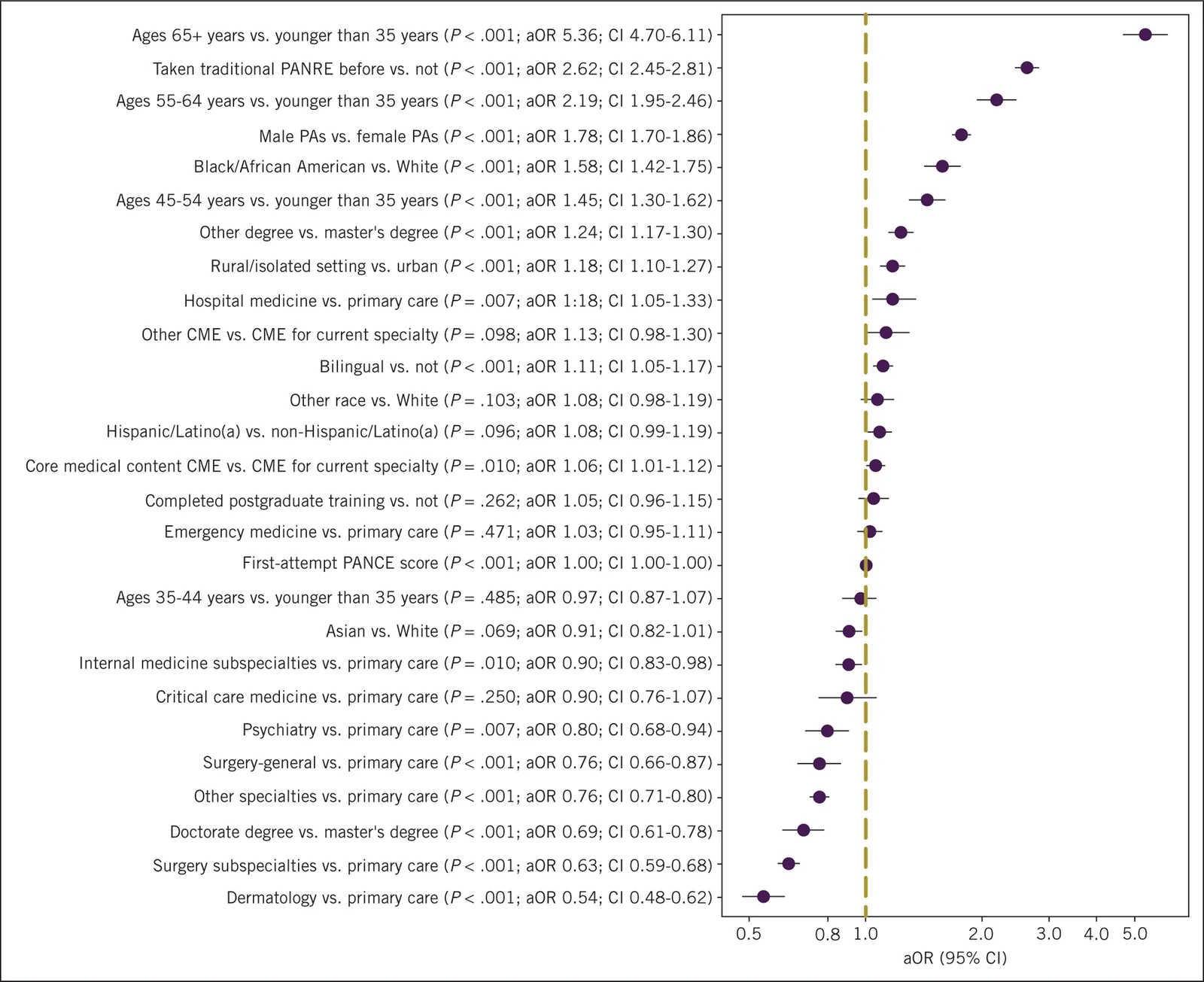
Factors associated with increased and decreased odds of choosing traditional PANRE over PANRE-LA

Multivariate logistic regression results showed that the decision to opt for the traditional PANRE was most strongly associated with older age, previous experience with the traditional PANRE, identifying as male, and identifying as African American/Black (Figure 5). PAs 65 years and older, compared with those younger than 35, had over five times higher odds of choosing the traditional PANRE (adjusted odds ratio [aOR] 5.36; *P* < .001). Having previously taken the traditional PANRE was associated with 2.62 times higher odds of choosing it again. Male PAs had 78% higher odds (aOR 1.78; *P* < .001) of choosing the traditional PANRE than female PAs, and African American/Black PAs, compared with their White colleagues, had 58% higher odds (aOR 1.58; *P* < .001) of selecting the traditional PANRE. Other factors associated with increased odds of choosing the traditional PANRE over the PANRE-LA included having completed a degree below a master's level (PA certificate, associate, bachelor's) versus having completed a master's degree (aOR 1.24; *P* < .001); residing in a rural/isolated area versus an urban area (aOR 1.18; *P* < .001); and practicing in hospital medicine versus primary care (aOR 1.18; *P* = .007). Conversely, the lowest odds of selecting the traditional PANRE were observed among PAs practicing in dermatology (aOR 0.54; *P* < .001) and surgery subspecialties (aOR 0.63; *P* < .001), relative to those in primary care, and among PAs with a doctorate compared with those with a master's degree (aOR 0.69; *P* < .001).

## DISCUSSION

We explored demographic, practice, and prior board certification factors potentially associated with choosing the traditional PANRE over the PANRE-LA among a large cohort of PAs who were due for recertification. Overall, 77.6% chose the PANRE-LA. Despite the overall predominance of PANRE-LA selection, older age, male gender, and African American/Black race were strongly associated with traditional PANRE selection. Specialty also emerged as an important factor, with significant variation in the odds of selecting the traditional examination across practice areas. Prior experience with the traditional PANRE was a strong predictor of choosing it again. Performance on the most recent traditional PANRE and the initial-attempt PANCE score, however, showed only a weak association with examination format decision.

Among the factors assessed, older age was the strongest predictor of choosing the traditional PANRE over the PANRE-LA. This finding aligns with a study reporting that family medicine physicians who chose the FMCLA tended to be younger than those opting for the traditional 1-day examination.^17^ Another study found that younger pediatricians (those aged 30 to 39 years) had more favorable perspectives of their LA pilot, the Maintenance of Certification Assessment for Pediatrics (MOCA-Peds), than older pediatricians (those age 60 years and older).^16^ A recent study of the PANRE-LA found no significant differences in self-reported learning benefits among PAs by age.^9^

A likely explanation for the pronounced relationship between older age and selection of the traditional PANRE may be familiarity with the examination format. With older age, PAs may gain experience with the traditional PANRE, establish effective study strategies, and become familiar with the testing center environment. This familiarity may increase confidence by reducing perceived uncertainty about the testing experience. The finding that prior PANRE experience was the second strongest predictor of choosing the traditional PANRE further reinforces this interpretation. PAs who have previously passed the traditional PANRE may feel more confident replicating the steps needed to succeed again rather than switching to an unfamiliar format. Technology readiness may also play a factor in PAs' decision-making, as the PANRE-LA is delivered online over 2 to 3 years, requiring reliable internet and frequent interaction with the online platform.

Qualitative research is needed to further explore the observed age-related differences in PAs' choice of recertification examination. This is particularly notable as research shows that performance on summative traditional assessments tends to decrease with older age or years in practice.^22^-^24^ This trend was also observed in the present study, with mean scores on the most recent traditional PANRE declining slightly with older age. Older providers and those further from their initial training may benefit more from LAs, which are designed to promote continuous learning.

Another significant finding was the difference in examination selection by gender, whereby male PAs were more likely to choose the traditional PANRE. This association was consistently observed across the overall bivariate, subgroups (by age and prior traditional PANRE experience), and multivariate analyses. This is consistent with one study's finding that male family physicians were more likely to choose the 1-day examination.^17^ Other research suggests potential gender differences among physicians in test-taking confidence, which may be relevant for interpreting our findings. Another study found that male physicians participating in the American Board of Physical Medicine and Rehabilitation's LA pathway reported higher confidence in their responses than their female counterparts, even when their answers were incorrect.^25^ This difference in confidence may have contributed to the patterns of examination-type selection observed in our study. Male PAs may favor the traditional PANRE if they perceive a lesser need for the formative components available in the PANRE-LA. Further research is required to test this potential explanation.

Our study also identified racial differences in examination choice, with African American/Black PAs demonstrating a greater likelihood of selecting the traditional PANRE than other racial groups. Prior research exploring PAs' perceptions of the value of certification showed that African American/Black PAs generally held more favorable views across several dimensions—such as that certification helps update medical knowledge, helps monitor or improve the quality of patient care, and helps to advance PAs professionally—than White PAs.^26^ Furthermore, qualitative data from the same study suggested that PAs from diverse backgrounds perceived certification to be important for professional validation in the workplace, particularly in relation to racial discrimination. African American/Black PAs may perceive the traditional PANRE as a more rigorous assessment, conferring a stronger sense of professional validation than the open-book PANRE-LA format. However, other research that focused on PANRE-LA participants found that African American/Black PAs held equally positive perceptions of the PANRE-LA as PAs from other racial groups.^9^ Further investigation is warranted to reconcile these findings.

Other factors were significantly associated with traditional PANRE selection, although their associations were less pronounced than those previously discussed. Having completed a degree below a master's level (PA certificate, associate, or bachelor's) versus having completed a master's degree was associated with increased odds of choosing the traditional PANRE; this is likely attributed to PAs with degrees below a master's level being older and having more experience with the traditional examination. Residing in rural/isolated areas was also significantly associated with choosing the traditional PANRE, potentially reflecting concerns about stable internet connectivity necessary for the PANRE-LA platform. Furthermore, those working in hospital medicine were the only PAs, when compared with primary care PAs, with higher odds of choosing the traditional PANRE over the PANRE-LA. PAs in hospital medicine are among the youngest practicing PAs with a median age of 36, and 19.4% are under 30 years old.^27^ This finding contrasts our other finding that younger age was strongly associated with selecting the PANRE-LA. These findings also require further exploration through qualitative research.

Although differences in prior examination performance between the traditional PANRE and the PANRE-LA groups were statistically significant, their magnitude was small, suggesting limited practical significance. For example, although PAs who chose the traditional PANRE scored statistically higher on their first-attempt PANCE scores than those who selected the PANRE-LA, the mean difference in scores was only 7.9 points. Similarly, where age-stratified subgroup differences were significant (ages 35 to 44, 45 to 54, 55 to 64, and 65 years and older), the point differences for the traditional PANRE group versus the PANRE-LA group (ranging from 5.8 to 13.1 points) do not translate to practically meaningful differences in performance.

Similarly, the findings for differences in the most recent traditional PANRE performance, while demonstrating Simpson's paradox, highlighted even smaller score differences.^21^ The overall analysis revealed a statistically significantly higher mean score for the PANRE-LA group compared with the traditional PANRE group (509.1 vs 504.7; *P* < .001). The age-stratified analyses which revealed the paradox, however, showed that within most age groups (ages 35 to 44, 45 to 54, 65 years and older), PAs who chose the traditional PANRE scored marginally higher, with mean differences ranging from just 2.8 to 5.6 points compared with PAs who chose the PANRE-LA. This reversal is attributed to age acting as a confounding factor, given that traditional PANRE performance slightly declines with older age, and younger PAs, who tend to perform better, were more likely to select the PANRE-LA, which influenced the overall mean comparison. Despite the statistical significance driven by the large sample size, the actual score differences observed among PAs who chose the traditional PANRE versus those who chose the PANRE-LA appear negligible.

We also identified factors associated with a decreased likelihood of choosing the traditional PANRE. PAs practicing in more specialized disciplines such as dermatology and surgery subspecialties had lower odds of selecting the traditional PANRE than their colleagues in primary care. One possible reason is that PAs in specialized fields may choose the PANRE-LA for its formative elements, which offer ongoing opportunities to reinforce and update core medical knowledge. PAs in specialized disciplines may utilize broader foundational knowledge less frequently in daily practice. Maintaining up-to-date core medical knowledge, however, remains important, as career flexibility is a hallmark of the PA profession: 76% of PAs transition between specialties at least once in their careers.^28^

Lastly, PAs with a doctorate were less likely to choose the traditional PANRE than those with a master's degree. Pursuing and earning a doctorate reflects a commitment to ongoing professional development and lifelong learning. PAs who have completed a doctorate may choose the PANRE-LA to participate in continuous learning. More research is needed to confirm this interpretation.

### Limitations

We did not assess self-reported reasons for choosing one assessment over the other; instead, we relied on existing data to analyze factors potentially associated with PAs' choices. PAs who chose the traditional PANRE may prefer a one-and-done assessment over the PANRE-LA, which requires sustained engagement over multiple years. Another potential reason we did not assess was whether technology barriers, such as internet connectivity, were a determining factor in PAs' choices. Additionally, the present analysis did not account for the possibility that the traditional PANRE is the default assessment option, and some PAs may have missed the deadline to apply for the PANRE-LA, rather than explicitly choosing the traditional PANRE. There may also be differences among PAs engaged in nonclinical work, such as primary educational roles, which we did not assess.

## CONCLUSION

A substantial majority of PAs assessed in this study opted for the PANRE-LA to fulfill their recertification requirements. However, older PAs showed a greater tendency to select the traditional PANRE format. Demographic factors such as identifying as male and as African American/Black were also associated with choosing the traditional PANRE. Prior experience with the traditional PANRE emerged as a strong predictor of choosing it again. In contrast, performance on recent PANRE administrations and initial PANCE attempt scores demonstrated only a weak association with examination selection. Although our findings highlight demographic and experiential differences in recertification examination selection, the underlying motivations remain unclear. Further qualitative research is warranted to explore PAs' self-reported reasons for their decisions. Understanding these factors is crucial for meeting credentialing expectations and promoting equitable access to preferred assessment options.

## References

[1] ABMS Insights. All ABMS Member Boards Now Offer Formative Assessments. April 4, 2023. Accessed August 27, 2024. https://www.abms.org/newsroom/all-abms-member-boards-now-offer-formative-assessments/#:~:text=Seven%20of%20the%2017%20Member,across%20the%20seven%20Member%20Boards

[2] American Board of Internal Medicine. Longitudinal Knowledge Assessment. 2024. Accessed August 27, 2024. https://www.abim.org/maintenance-of-certification/assessment-information/assessment-options/longitudinal-knowledge-assessment/

[3] American Board of Family Medicine. Continue Certification. 2024. Accessed August 27, 2024. https://www.theabfm.org/continue-certification/exam/

[4] The American Board of Pediatrics. The Four Parts of MOC. 2024. Accessed August 27, 2024. https://www.abp.org/content/four-parts-moc

[5] Roswell RO Johnson EN Jain R. Maintenance of certification—the value to patients and physicians. *JAMA*. 2024;331(9):727–728. doi:10.1001/jama.2024.0374 38315157 10.1001/jama.2024.0374

[6] Newton WP Rode K O'Neill T Fain R Baxley E Peterson L. Family medicine certification longitudinal assessment after one year. *J Am Board Fam Med*. 2020;33(2):344–346. doi:10.3122/jabfm.2020.02.190055

[7] Newton WP Baxley E O'Neill TR Rode K Fain R Stelter KL. Family medicine certification longitudinal assessment becomes permanent. *J Am Board Fam Med*. 2021;34(4):879–881. doi:10.3122/jabfm.2021.04.210242

[8] The American Board of Pediatrics. MOCA-Peds: A Review of Participation Since 2019. 2024. Accessed September 4, 2024. https://www.abp.org/dashboards/moca-peds-participation-since-2019

[9] Kozikowski A Goodman J Dallas A Jiang Y. Equity and longitudinal assessments: perspectives from physician assistants/associates (PAs) participating in PANRE-LA. *Med Sci Educ*. 2025;35(3):1461–1472. doi:10.1007/s40670-025-02329-4 40625970 10.1007/s40670-025-02329-4 PMC12228929

[10] Demeter JM. Recertification through longitudinal assessment for PAs. *JAAPA*. 2025;38(4):41–44. doi:10.1097/01.JAA.0000000000000099 10.1097/01.JAA.0000000000000099 40130914

[11] Rottman BM Caddick ZA Nokes-Malach TJ Fraundorf SH. Cognitive perspectives on maintaining physicians' medical expertise: I. Reimagining Maintenance of Certification to promote lifelong learning. *Cogn Res Princ Implic*. 2023;8(1):46. doi:10.1186/s41235-023-00496-9 37486508 10.1186/s41235-023-00496-9 PMC10366070

[12] Caddick ZA Fraundorf SH Rottman BM Nokes-Malach TJ. Cognitive perspectives on maintaining physicians' medical expertise: II. Acquiring, maintaining, and updating cognitive skills. *Cogn Res Princ Implic*. 2023;8(1):47. doi:10.1186/s41235-023-00497-8 37488460 10.1186/s41235-023-00497-8 PMC10366061

[13] Ward RC Baker KA Spence D Leonard C Sapp A Choudhry SA. Longitudinal assessment to evaluate continued certification and lifelong learning in healthcare professionals: a scoping review. *Eval Health Prof*. 2023;46(3):199–212. doi:10.1177/01632787231164381 36961523 10.1177/01632787231164381

[14] Robinson LR Raddatz MM Kinney CL. Evaluation of longitudinal assessment for use in maintenance of certification. *Am J Phys Med Rehabil*. 2020;99(5):420–423. doi:10.1097/PHM.0000000000001359 31809270 10.1097/PHM.0000000000001359

[15] Choudhry SA Muckle TJ Gill CJ Transforming assessments of clinician knowledge: a randomized controlled trial comparing traditional standardized and longitudinal assessment modalities. *Pract Assess Res Eval*. 2024;29(7):1–21. Accessed July 28, 2026. https://openpublishing.library.umass.edu/pare/article/id/2028/

[16] Turner AL Olmsted M Smith AC Pediatrician perspectives on learning and practice change in the MOCA-Peds 2017 pilot. *Pediatrics*. 2019;144(6):e20192305. doi:10.1542/peds.2019-2305 31690712 10.1542/peds.2019-2305

[17] Virani M Fleischer S Peterson LE. Changes in family medicine certification examination performance in longitudinal assessment. *J Contin Educ Health Prof*. 2025;45(4):226–232. doi:10.1097/CEH.0000000000000606 40340967 10.1097/CEH.0000000000000606

[18] Glicken AD. Physician assistant profile tool provides comprehensive new source of PA workforce data. *J Am Acad Physician Assist*. 2014;27(2):47–49. doi:10.1097/01.JAA.0000442708.26094.02 10.1097/01.JAA.0000442708.26094.02 24463752

[19] Glicken AD Miller AA. Physician assistants: from pipeline to practice. *Acad Med*. 2013;88(12):1883–1889. doi:10.1097/ACM.0000000000000009 24128623 10.1097/ACM.0000000000000009

[20] O'Brien RM. A caution regarding rules of thumb for variance inflation factors. *Qual Quant*. 2007;41(5):673–690. doi:10.1007/s11135-006-9018-6

[21] Samuels ML. Simpson's paradox and related phenomena. *J Am Stat Assoc*. 1993;88(421):81–88. doi:10.1080/01621459.1993.10594297

[22] Marco CA Wahl RP House HR Physician age and performance on the American Board of emergency medicine concert examination. *Acad Emerg Med*. 2018;25(8):891–900. doi:10.1111/acem.13420 29608798 10.1111/acem.13420

[23] Driscoll SW Geis CC Raddatz MM Kinney CL Robinson LR. Predictors of performance on the American Board of Physical Medicine and Rehabilitation maintenance of certification examination. *PM R*. 2018;10(12):1361–1365. doi:10.1016/j.pmrj.2018.06.009 29964209 10.1016/j.pmrj.2018.06.009

[24] Sun H Culley DJ Lien CA Kitchener DL Harman AE Warner DO. Predictors of performance on the Maintenance of Certification in Anesthesiology Program^®^ (MOCA^®^) examination. *J Clin Anesth*. 2015;27(1):1–6. doi:10.1016/j.jclinane.2014.08.007 25468588 10.1016/j.jclinane.2014.08.007

[25] Kinney CL Raddatz MM Robinson LR. Influence of sex and age on ratings of confidence and relevance in continuing certification longitudinal assessment: a pilot study. *Am J Phys Med Rehabil*. 2021;100(2S):S3‖S6. doi:10.1097/PHM.0000000000001635 33141772 10.1097/PHM.0000000000001635

[26] Kozikowski A Morton-Rias D Puckett K Jeffery C Mauldin S Goodman J. The association of physician assistant/associate demographic and practice characteristics with perceptions of value of certification. *BMC Med Educ*. 2023;23(1):228. doi:10.1186/s12909-023-04215-2 37041511 10.1186/s12909-023-04215-2 PMC10088160

[27] National Commission on Certification of Physician Assistants. *2023 Statistical Profile of Board Certified PAs by Specialty: An Annual Report of the National Commission on Certification of PAs*. 2024. Accessed May 1, 2025. https://www.nccpa.net/wp-content/uploads/2024/07/2023-Statistical-Profile-of-Board-Certified-PAs-by-Specialty-Annual-Report.pdf#page=69.04

[28] Kozikowski A Bruza-Augatis M Morton-Rias D Physician assistant/associate career flexibility: factors associated with specialty transitions. *BMC Health Serv Res*. 2024;24(1). doi:10.1186/s12913-024-12158-7 10.1186/s12913-024-12158-7 PMC11681730 39732670

